# Novel insight into mechanisms for ATR activation by chromatin structures

**DOI:** 10.1007/s00204-021-03133-w

**Published:** 2021-08-11

**Authors:** Al-Hassan M. Mustafa, Oliver H. Krämer

**Affiliations:** 1 Institute of Toxicology, Mainz University Medical Center, Mainz, Germany; 2 Department of Zoology, Faculty of Science, Aswan University, Aswan, Egypt

Sophisticated molecular networks ensure that human cells replicate about 3 × 10^9^ base pairs faithfully in each cell cycle. Disturbances in DNA replication, such as upon dNTP depletion by the clinically relevant ribonucleotide reductase inhibitor hydroxyurea, activate complex signaling cascades. The serine/threonine kinase ataxia–telangiectasia mutated and Rad3 related (ATR) is one of the first molecules that is activated when DNA replication halts. The ATR substrate single-strand DNA-binding protein RPA protects single-stranded DNA that arises when DNA helicases continuously open DNA, despite dNTP levels being too low due to hydroxyurea. Checkpoint kinase-1/-2 (CHK1/CHK2) and ataxia–telangiectasia mutated (ATM) are activated by ATR in cells with replication stress. The concerted action of these and further kinases and phosphatases arrests the cell cycle and modulates DNA repair if replication forks collapse and become DNA breaks (Williams and Zhang 2021).

The term chromatin describes DNA that is densely packed by histones and other positively charged proteins. To allow DNA repair and DNA replication, chromatin is biochemically altered by posttranslational modifications. These include the addition of acetyl and methyl residues to lysine residues. Zhu and colleagues elegantly show that the chromatin complex is far more than a passive element during DNA replication stress in human cancer cells (Zhu et al. 2021). They reveal that specifically the tri-methylation of histone H3 at lysine residue-14 (H3K14me3) by the histone methyltransferase SETD2 controls ATR activation and cell fate upon hydroxyurea-induced stress. To examine how H3K14 methylation affects cellular responses to stress, Zhu and colleagues generated novel antibodies that specifically recognize H3K14 mono-, di-, and tri-methylation. They found that hydroxyurea time- and dose-dependently increased the levels of H3K14me3 in cervix and colon-derived tumor cells. This occurred at the expense of mono- and di-methylated H3K14 and paralleled the accumulation of phosphorylated RPA. The amino acid sequence preceding the H3K14 site is reminiscent of the sequence before H3K36, a known substrate of SETD2. Therefore, Zhu and colleagues speculated that SETD2 methylates H3K14. Genetic elimination of SETD2 by RNAi and CRISPR-Cas9 verified that SETD2 is responsible for the basal and replication stress-induced accumulation of H3K14me3. Remarkably, this was associated with increased recruitment of SETD2 to chromatin and a loss of SETD2 suppressed the phosphorylation of ATR, RPA, and CHK1. Unbiased pulldowns with an H3K14me3 peptide and immuno-precipitations with the anti-H3K14me3 antibody identified an interaction of H3K14me3 with the RPA complex. Mutation analysis of H3K14 corroborated that this lysine residue is critical for a stress-induced recruitment of ATR, CHK1, and RPA to chromatin. Accordingly, cells lacking SETD2 or overexpressing H3K14 mutant molecules are more sensitive to cytotoxic effects of hydroxyurea and recover less efficiently from DNA replication stress upon drug removal.

Like any interesting novel insight, this work points to future research directions. Zhu and colleagues speculate that the levels of SETD2 and the tri-methylation of H3K14 allow stratifying patients into responders and non-responders to chemotherapeutics that target DNA replication. This corresponds to the notion that a pharmacological inhibition of ATR promotes the elimination of tumor cells with replication stress by excessive DNA damage and apoptotic cell death. Future research can decipher how SETD2 and its impact on H3K14 methylation regulate DNA damage upon DNA replication stress. Is this strictly through ATR or are there further mechanism involved? Since SETD2 promotes the expression of the ribonucleotide reductase subunit RRM2, the target of hydroxyurea, this mechanism may also regulate the sensitivity of cells to hydroxyurea. Moreover, SETD2 and histone methylation may contribute to additional signaling cascades that suppress the sensitivity of tumor cells to hydroxyurea (Pons et al. 2021). It will likewise be exciting to see how the complex between SETD2 and RPA is formed and how SETD2 is recruited to chromatin when the dNTP supply becomes limited. This mechanism is probably also engaged when oncogenes are activated during cell transformation and cause DNA hyper-replication. Furthermore, there might be signals that allow SETD2 to leave the chromatin upon drug removal. Post-translational modifications of SETD2 may control these processes in complex cross-regulatory pathways.

In sum, the work by Zhu and colleagues is an important step towards a better understanding how chromatin structure controls signaling cascades and cell fate upon DNA replication stress.
